# 

**Published:** 1853-03

**Authors:** 


VOL. I. NEW SERIES. No. 11.

### THE NORTH-WESTERN

### MEDICAL AND SURGICAL

### JOURNAL.

PUBLISHED MONTHLY,

AT TWO DOLLARS PER ANNUM.

EDITED BY

W. B. HERRICK, M.D.,

PROFESSOR OF ANATOMY AND PHYSIOLOGY IN RUSH MEDICAL COLLEGE; SURGEON TO THE U. S. MARINE HOSPITAL, ONE OF THE SURGEONS OF THE ILLINOIS GENERAL HOSPITAL, ETC.

ASSISTED BY

H. A. JOHNSON, M. D.

MARCH, 1853.

CHICAGO:

PUBLISHED BY BALLANTYNE & CO., PRINTERS & BOOKBINDERS.

NEW POST-OFFICE BUILDINGS, COR. OF CLARK AND RANDOLPH-STS., OPPOSITE THE SHERMAN HOUSE.

1853.

VOL I. WHOLE SERIES. No. 11.

